# The Standardization of the Wassermann Reaction

**Published:** 1919-01

**Authors:** 


***The Standardization of the Wassermann Reaction.*** By W. d'Este Emery. *The Lancet*, Oct. 26, 1918.

D'Este Emery believes that a method involving the use of crude serum is the best as a means for the diagnosis of syphilis. The problem he takes up is : " What strength of reaction is to be regarded as being necessary to diagnose syphilis? " In a just positive W. reaction there are three reacting substances in a state of equilibrium. Should more complement be added, it will remain free, but will be absorbed if more serum or more antigen is added.

The question is to titrate the unknown substance, the strength of which is to be determined by means of two standard solutions, the excess of either of which is compensated for (within certain limits) by the corresponding excess of the other.

The difficulty of the problem will be understood when it is said that reagents cannot be standardized by ordinary chemical or physical processes. There seems to be no starting point. How is it possible to obtain trustworthy results in actual practice? The author thinks it is largely a matter of personal equation on the part of the experimenter.

The first essential is for experimenters to agree on some unit, some standard to which all results can be compared; not that this uniform technic should be imposed in practice, but used when comparison is needed.

In his technic d'Este Emery uses a five per cent emulsion of sheep's corpuscles, measured in a graduated pipette; he does not centrifugalize to constant volume in a graduated tube. The standardization of the complement is carried out with the same emulsion.

In actual practice he uses 40 amboceptor units (of his own) per cc. He emphasizes that his units are not fixed units, but only relative one to the other. After the dosage of the amboceptor, according to Macintosh and Filde's method, he proceeds to the standardization of the complement, and afterwards to the preparation of the Antigen-complement mixture.

The author thinks that standardizing the complement in presence of the antigen enables one to state without hesitation the actual amount of complement used in the test. On the question of mixing the complement and antigen (both prepared of double strength) just before the test, and not making up a large volume of the test fluid for use throughout the test, he does not find there is any continued absorption of complement for the first two hours after the two fluids have been mixed.

He points out that preparing a standard Wassermann unit by the use of antigen composed in a definite manner is possible, and that the absorption of the two clear units of complement should be taken as a standard unit.

Antigen prepared after his manner remains remarkably constant; syphilitic sera, asepticised, remain also constant for over a month at room temperature, enabling the experimenter to repeat observation.

Too much stress can not be laid on the use of accurately standardized syphilitic sera since it secures a scale of comparison for experimenters. It would, of course, be important to agree on some definite unit, common to all laboratories for the Wassermann reaction.

